# Combination of Farnesol with Common Antifungal Drugs: Inhibitory Effect against *Candida* Species Isolated from Women with RVVC

**DOI:** 10.3390/medicina59040743

**Published:** 2023-04-10

**Authors:** Fatemeh Nikoomanesh, Mahsa Falahatinejad, Lucia Černáková, André Luis Souza dos Santos, Shahla Roudbar Mohammadi, Mitra Rafiee, Célia Fortuna Rodrigues, Maryam Roudbary

**Affiliations:** 1 Infectious Disease Research Center, Birjand University of Medical Sciences, Birjand 9717853577, Iran; 2 Department of Medical Mycology, Faculty of Medical Sciences, Tarbiat Modares University, Tehran 14115111, Iran; 3 Department of Microbiology and Virology, Faculty of Natural Sciences, Comenius University in Bratislava, 842 15 Bratislava, Slovakia; 4 Department of General Microbiology, Microbiology Institute Paulo de Góes, Federal University of Rio de Janeiro (UFRJ), Rio de Janeiro 21941-901, RJ, Brazil; 5 Department of Immunology, School of Medicine, Cellular and Molecular Research Center, Birjand University of Medical Sciences, Birjand 9717853577, Iran; 6 LEPABE—Laboratory for Process Engineering, Environment, Biotechnology and Energy, Faculty of Engineering, University of Porto, 4200-465 Porto, Portugal; 7 ALiCE—Associate Laboratory in Chemical Engineering, Faculty of Engineering, University of Porto, 4200-465 Porto, Portugal; 8 TOXRUN—Toxicology Research Unit, Cooperativa de Ensino Superior Politécnico e Universitário—CESPU, 4585-116 Gandra PRD, Portugal; 9 Department of Parasitology and Mycology, School of Medicine, Iran University of Medical Sciences, Tehran 1449614535, Iran

**Keywords:** vulvovaginal candidiasis, farnesol, azoles, resistance

## Abstract

*Background and Objectives*: Vulvovaginal candidiasis (VVC) is a mucous membrane infection, with an increased rate of antifungal resistance of *Candida* species. In this study, the in vitro efficacy of farnesol alone or in combination with traditional antifungals was assessed against resistant *Candida* strains recovered from women with VVC. *Materials and Methods*: Eighty *Candida* isolates were identified by multiplex polymerase chain reaction (PCR), and the antifungal susceptibility to amphotericin B (AMB), fluconazole (FLU), itraconazole (ITZ), voriconazole (VOR), clotrimazole (CTZ), and farnesol was tested by the standard microdilution method. The combinations of farnesol with each antifungal were calculated based on the fractional inhibitory concentration index (FICI). *Result: Candida glabrata* was the predominant species (48.75%) isolated from vaginal discharges, followed by *C. albicans* (43.75%), *C. parapsilosis* (3.75%), a mixed infection of *C. albicans* and *C. glabrata* (2.5%) and *C. albicans* and *C. parapsilosis* (1%). *C. albicans* and *C. glabrata* isolates had lower susceptibility to FLU (31.4% and 23.0%, respectively) and CTZ (37.1% and 33.3%, respectively). Importantly, there was “synergism” between farnesol–FLU and farnesol–ITZ against *C. albicans* and *C. parapsilosis* (FICI = 0.5 and 0.35, respectively), reverting the original azole-resistant profile. *Conclusion*: These findings indicate that farnesol can revert the resistance profile of azole by enhancing the activity of FLU and ITZ in resistant *Candida* isolates, which is a clinically promising result.

## 1. Introduction

Vulvovaginal candidiasis (VVC) is a female major mucous membrane infection of the lower genital tract, caused by *Candida* species, and associated with an increased rate of antifungal resistance, with severe or long-term daily symptoms [1,2,3,4]. Per year, approximately 40 to 50% of women have a history of VVC infection, and around 138 million women develop recurrent vulvovaginal candidiasis (RVVC). The symptoms of both recurrent and acute vaginal candidiasis are mostly similar. In developing countries such as Iran, the prevalence of RVVC is >4300 cases per 100,000 women [5,6]. A recent study has confirmed that Iran is among the countries with the highest rate of RVVC, and *C. albicans* and *C. glabrata* are the most common agents [7].

The prevalence of VVC infections has significantly increased, probably related to the extensive use of azoles for both prophylactic and therapeutic purposes [8]. Azoles are the first-choice drugs for the initial treatment of VVC, but the long-term use of fluconazole has resulted in the development of multidrug-resistant (MDR) and recurrent infections, which is a critical healthcare problem. Due to some limitations related to the availability of certain antifungal drugs, inefficient treatment, high toxicity, low tolerability, and drug interaction, the search for new compounds with antifungal properties is an urgent necessity to overcome the drug resistance problem [9].

Farnesol (C_15_H_26_O), a sesquiterpene alcohol that was first described as a *quorum-sensing* molecule produced in *C. albicans*. It has attracted high attention regarding its pharmacological activities, such as antitumor, antioxidant, and antimicrobial properties [10,11,12,13]. Some studies have reported that farnesol inhibits hyphae formation, also inhibiting drug transporters [14,15,16,17]. Published data have proposed that farnesol has in vitro synergistic effects with various antifungals such as nystatin, itraconazole, fluconazole, amphotericin B, and echinocandins [18,19]. Furthermore, this signaling molecule was observed to inhibit the biofilm formation, and in combination with certain antifungals, farnesol can even serve as an adjuvant in the therapy of candidiasis [20]. For this purpose, combination antifungal therapy is considered a promising strategy, especially against resistant *Candida* isolates, increasing the effectiveness of common antifungal agents and reverting antifungal resistance among clinical isolates [21,22].

Hence, this study aimed to identify the *Candida* species recovered from women suffering from VVC and determine the susceptibility of resistant *Candida* isolates against antifungal drugs and farnesol—alone and in combination.

## 2. Materials and Methods

### 2.1. Sample Collection

Eighty *Candida* isolates were recovered from 150 women with VVC from the obstetrics and gynecology wards, Birjand University of Medical Sciences (Iran), between the period of December 2018 and March 2019. This study was approved by the ethics committee of the Iran University of Medical Sciences, Iran (no. 1399.921). All participants signed a written consent form before participating in the study.

### 2.2. Identification of Isolates

#### 2.2.1. Conventional Methods

All isolates were examined for direct examination to detect yeasts and pseudohyphae and then cultured on Sabouraud dextrose agar (SDA; Merck, Darmstadt, Germany) for viability and purity. After the sample collection, the primary identification was carried out using conventional methods, such as a germ tube test, chlamydospore formation (cornmeal agar test), and CHROMagar *Candida* medium (CHROMagar™, Sigma-Aldrich, St. Louis, MO, USA) for 48 h at 35 °C [23].

#### 2.2.2. Molecular Assay

For the multiplex polymerase chain reaction (PCR) assay [24], genomic DNA was extracted using the acetyl trimethylammonium bromide-based method previously described [25]. Species-specific primers [26] were used (Table 1) for the precise identification of each *Candida* species. PCR products were analyzed on 2% agarose gel electrophoresis and checked visually by Gel Doc (Gel Doc XR+, Bio-Rad, Hercules, CA, USA). The identification of *Candida* species was performed by comparison of the sizes of the fragments with the references’ band profiles (Table 1). Moreover, *C. albicans* (ATCC 10231), *C. parapsilosis* (ATCC 22019), and *C. glabrata* (ATCC 2001) were used for standard strains. All the experiments were carried out in duplicate.

### 2.3. Antifungal Susceptibility Testing 

The antifungal susceptibility testing (AFST) of *Candida* isolates were performed against amphotericin B (AMB), itraconazole (ITZ), voriconazole (VOR), fluconazole (FLU), and clotrimazole (CTZ) (Sigma-Aldrich, Oakville, ON, Canada) using the standard broth microdilution method and according to the Clinical and Laboratory Standards Institute (CLSI M27-A3/S4) guidelines [27]. Briefly, dilutions were prepared in RPMI-1640 medium (Roswell Park Memorial Institute; Sigma Chemical Co., St. Louis, MO, USA) in 96-well flat-bottom microtiter plates (Nunc^TM^, Thermo Fisher Scientific, Illkirch-Graffenstaden, France). For AMB, ITZ, VOR, and CTZ, the concentrations ranged from 0.016 to 16 μg/mL, whereas for FLU, it ranged from 0.063 to 64 μg/mL. Each *Candida* isolate was inoculated at a concentration of 0.5–2.5 × 10^3^ CFU/mL and incubated at 35 °C for 24 h. *C. albicans* (ATCC 10231), *C. parapsilosis* (ATCC 22019), and *C. krusei* (ATCC 6258) were used for quality control purposes. The minimum inhibitory concentrations (MICs) were defined as at least 50% and 90% growth reduction (azoles and AMB respectively) compared with the untreated control. The growth of fungi in the wells was checked visually.

Lastly, *Candida* isolates were categorized as susceptible and resistant to:

- FLU: MIC ≥ 8 μg/mL—resistance; MIC ≤ 2 μg/mL—susceptible, and MIC = 4 μg/mL—8 μg/mL dose-dependent susceptibility;
- Other azoles: MIC ≤ 0.12 μg/mL—susceptible and MIC ≥ 1 μg/mL—resistant;
- AMB: MIC ≤ 2 μg/mL—susceptible and MIC > 2 μg/mL—resistant.

All tests were performed in three independent experiments and repeated at least three times.

### 2.4. Antifungal Activity of Farnesol

The antifungal activity of farnesol (Sigma-Aldrich, Klongton, Klongtoey, Thailand) on *Candida* proliferation was performed as previously reported, with a minor modification [28]. For this, farnesol was diluted with methanol, to obtain a stock solution at a concentration of 30 mM (10 µL of farnesol was added to 1 mL 10% methanol). Subsequently, the farnesol stock solution was adjusted to a concentration of 300 µM. For the in vitro proliferation assay, a concentration of 10^3^ *Candida* cells/mL were inoculated in a yeast nitrogen base (YNB, Sigma-Aldrich, New Jersey, NJ, USA) medium supplemented with farnesol at different final concentrations (5, 10, 20, 50, 100, 150, and 300 µM) in 96 well-microplates and incubated at 35 °C for 24 h. Negative and positive controls were prepared with farnesol-free and AMB, respectively. After this period, the rate of growth was determined by measuring the optical density (OD) absorption at 630 nm (Starsate, Germany). The MIC of farnesol was defined in comparison with the farnesol-free control.

### 2.5. Drug Combination Study

Combinations of farnesol and antifungals (FLU, AMB, ITZ, VOR, and CTZ) were tested against resistant isolates based on the CLSI (M27-A3/S4) protocol. The fractional inhibitory concentration index (FICI) was calculated to assess the drug interactions [29] using the following equation:

FICI = FIC (A) + FIC (B) = (MIC A combination/MIC A alone) + (MIC B combination/MIC of B alone).
(1)

Farnesol and antifungal interactions were classified as synergism—FICI ≤ 0.5, antagonism—FICI > 4.0, and indifferent—0.5 < FICI ≤ 4.0.

### 2.6. Cytotoxicity Assay

The cytotoxicity effects of farnesol on the SW480 cell line were carried out according to the Bio vision protocol [30]. In brief, the SW480 cell line was seeded (1 × 10^6^ cell/mL) in a 96-well microtiter plate with RPMI-1640 supplemented by fetal bovine serum 10% (FBS; Sigma-Aldrich, St. Louis, MO, USA) and incubated at 37 °C, 5% CO_2_, 90% humidity. Then, the cells were treated with 300 µM of farnesol, and untreated cells were considered as a control group. After 24 h, cells were harvested, washed, and 5 × 10^5^ cells/mL were transferred to a tube and resuspended in 100 μL of binding buffer. Then, 5 μL of FITC-conjugated annexin V (annexin V–FITC) and 5 μL of propidium iodide (PI) were added, incubated (15 min at room temperature, dark room), and analyzed by flow cytometer (Calibur, Becton Dickinson, Franklin Lakes, NJ, USA).

### 2.7. Statistical Analysis

Statistical analyses were performed using one-way analysis of variance (ANOVA) and *t*-tests using SPSS software version 20 (SPSS, Chicago, IL, USA). For all statistical analyses, *p* < 0.050 was considered statistically significant.

## 3. Results

**Figure 1:**
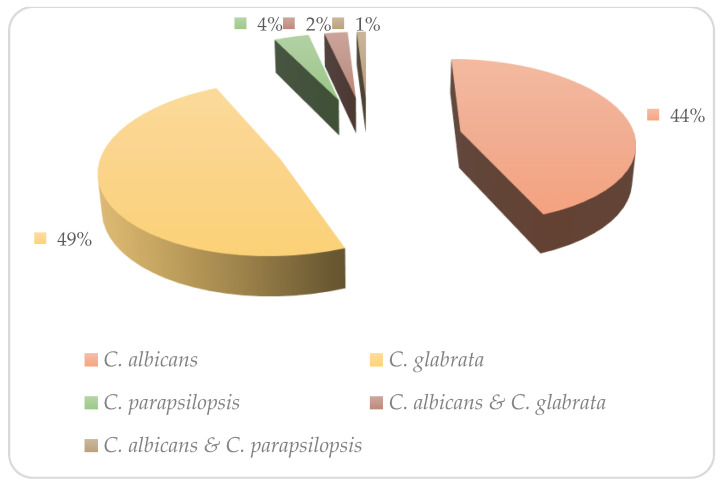
Prevalence of *Candida* species recovered from Iranian VVC patients.

Three species of *Candida* were presumptively identified in CHROMagar *Candida* medium and, subsequently, confirmed by PCR. *C. glabrata* was the predominant species (n = 39; 48.75%), followed by *C. albicans* (n = 35; 43.75%) and *C. parapsilosis* (n = 3; 3.75%). In addition, two mixed infections were detected using PCR, including *C. albicans* and *C. glabrata* (n = 2; 2.5%), *C. albicans,* and *C. parapsilosis* (n = 1; 1%) (Figure 1).

The results on the susceptibility assays of *Candida* species revealed that the highest resistance rates were detected against both FLU (65%) and CTZ (66%), particularly related to *C. albicans* (65.7%) and *C. glabrata* (71.8%). Importantly, all *C. albicans* isolates were susceptible to AMB, and all three *C. parapsilosis* were susceptible to ITZ (Table 2). For the *C. albicans* isolates, the MIC values of FLU, ITZ, VOR, and CTZ were interpreted based on the clinical breakpoints (CBP), while the AMB MIC values were evaluated based on the epidemiological cut-off values (ECV), due to a lack of information associated with CBP in CLSI for this drug.

The MIC concentrations varied greatly, depending on the compound: farnesol—150–300 µM; AMB—4–0.06 µg/mL, FLU 64–0.125 µg/mL, ITZ 4–0.06 µg/mL, VOR 6–0.125 µg/mL, and CTZ 8–0.06 µg/mL. Interestingly, a synergistic effect was observed in the combination of farnesol with FLU and farnesol and ITZ against *C. albicans* and *C. parapsilosis* isolates (FICI: 0.5 and 0.35, 0.25, respectively). The combination of VOR and AMB with farnesol against *C. parapsilosis* showed synergy (FICI: 0.5 and 0.35, respectively). In contrast, *C. glabrata* isolates showed no synergistic effect with any of the antifungal drugs and farnesol. Additionally, the MIC value of most drugs in combination with farnesol noticeably decreased in the resistant species: FLU from 8–64 to 2–8 µg/mL, ITZ from 1–8 to 1–4 µg/mL, VOR from 16–2 to 1–4 µg/mL, and AMB from 2 to 1 µg/mL. The combination of CTZ with farnesol did not show any effect. The results are shown in Table 3.

**Figure 2:**
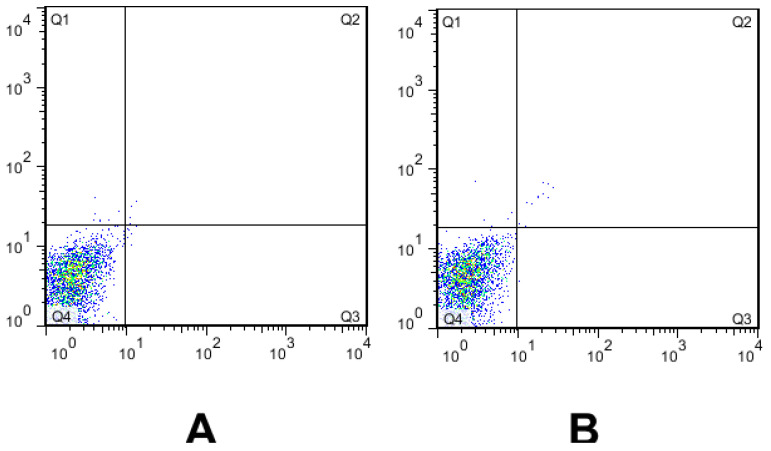
Apoptosis assay of the SW480 cell line when treated with 300 µM farnesol. Staining: annexin V and propidium iodide (PI). (**A**) Untreated cells (without farnesol, control group) and (**B**) treated cells with farnesol (Q1: viable cells, Q2: early apoptotic cells, Q3: late apoptotic cells and necrotic cells, and Q4: necrotic cells).

The cytotoxicity effect of farnesol on SW480 cells was carried out using a flow cytometry-based assay. According to Plots A (untreated SW480 cells—without farnesol as a control group) and Plots B (treated SW480 cells—with farnesol), farnesol did not show significant cytotoxic effects on the SW480 cell line at a concentration of 300 µM (Figure 2) (*p* > 0.05).

## 4. Discussion

VVC is one of the most common infections of the genital tract and a major worldwide concern in women’s health [31]. In this study, *C. glabrata* was the most isolated species from women suffering from VVC, followed by *C. albicans* and *C. parapsilosis.* This result was in agreement with other studies [4,32]. Indeed, we have witnessed a change in the prevalence of *Candida* species in candidiasis infections. Although *C. albicans* is still the dominant species in *Candida* infections, non-*albicans Candida* species (e.g., *C. parapsilosis* and *C. glabrata*) have been reported in isolates or in mixed infections with *C. albicans* [33,34,35]. By means of PCR assay, we were able to identify the mixed infections that, by CHROMagar, could not be distinguished with 100% certainty.

As expected, and according to the AFST findings, the *Candida* isolates showed different patterns of susceptibility to azoles. *C. albicans* and *C. glabrata* revealed a high rate of resistance to FLU, followed by CTZ, whereas approximately only 10% of *C. glabrata* isolates were resistant to AMB. In general, *Candida* isolates showed lower MIC against ITZ and AMB compared with FLU and VOR. Several other reports have highlighted the resistance to azole drugs in *Candida* species recovered from VVC, particularly to FLU [36,37,38,39]. In a work conducted by Bitew et al., 17.2% of *C. krusei* isolated from the vaginal tract were resistant to FLU [40], and Arastehfar et al. showed the high rate of FLU-resistant and FLU-tolerant phenotypes in *C. albicans* strains recovered from Iranian women suffering from VVC and RVVC [41]. Likewise, previous studies described a high percentage of *C. albicans* (81.5%) and *C. glabrata* (83.5%) recovered from Iranian pregnant women as FLU-resistant [42].

Despite being the first-line azole drug for the treatment of VVC, FLU susceptibility has significantly decreased in the last decades, due to the development of various mechanisms of resistance [43]. As a result, the search for efficient antifungal agents with minimum side effects and low toxicity is highly recommended [44,45]. Farnesol is a molecule synthesized by *C. albicans* via the enzymatic dephosphorylation of farnesyl pyrophosphate [46]. Farnesol inhibits *Candida* hyphae production in a concentration-dependent manner. Due to the noticeable inhibitory effects of farnesol on fungal cells, as well as antifungal activity, it has gained importance as a promising antifungal agent in recent decades [16,47,48]. Hence, in this study, the isolates previously found to be resistant were tested with the combination of farnesol and drugs. Our findings importantly showed that the combination of farnesol with antifungal drugs (farnesol–VOR and farnesol–AMB) had a synergism effect against clinical isolates of *C. albicans* and *C. parapsilosis* previously found to be resistant. This outcome clearly emphasizes the potential importance of farnesol as an effective antifungal agent. Similarly, Nagy et al. [49] showed synergy between triazoles and farnesol for three *C. auris* strains and one standard *C. albicans* in biofilms. Another study indicated that combining farnesol with FLU for FLU-resistant isolates partially increased the FLU activity, but this combination was not beneficial for susceptible isolates in the VVC model [50]. Moreover, Decanis et al. [51] showed farnesol was able to significantly decrease the Sap2 secretion, downregulated *Sap4–6* mRNA expression, and change yeast into hyphae morphogenesis in a *C. albicans* strain. It is important to note that several of these studies are in biofilms, and the present are in planktonic cells, so distinctive results are expected.

According to the drug susceptibility pattern of *C. glabrata*, these isolates have shown resistance to AMB in some cases. Therefore, these isolates with high resistance have been attributed no synergy. Rodrigues et al. [52] also tried combining two common antifungal drugs (AMB and posaconazole), and the FICI showed that the combination did not bring a clear advantage for this species. However, once more, the study was performed in biofilms [52]. Additionally, in agreement with our results, other studies notably confirmed the antifungal effects of farnesol against *Candida* species. For example, Cordeiro et al. [53] indicated that a combination of farnesol (ranged from 4.68 to 150 µM) with traditional antifungals significantly reduced the MICs of antifungals (FLU, ITZ, AMB, and caspofungin) against drug-resistant *Candida* species. In addition to studies on the combination of farnesol and traditional antifungals on planktonic cells, the study of the synergism of farnesol–antifungals in reducing the biofilm formation of *Candida* isolates has also been investigated. Another study reported a synergistic effect between farnesol and FLU/5-fluorocytosine, as it reduced the capacity for biofilm formation [54]. Furthermore, liposomal farnesol potentiated the action of FLU against *C. albicans* and *C. tropicalis*, but the association of unconjugated farnesol with FLU resulted in antagonistic effects [55]. Additionally, Katragkou et al. [18] found a synergistic or additive effect between farnesol and FLU, AMB, and micafungin in *C. albicans* biofilms.

In our study, farnesol has been described as a nontoxic drug at a concentration of 300 µM when synergized with azoles. This interaction leads to reactive oxygen species accumulation (which triggers apoptosis cell death) and influences drug extrusion, resulting in a shift of MIC [56]. Additionally, farnesol is able to modulate the activity of ABC efflux transporters, which can result in changes in the susceptibility profile to azoles in *C. albicans* or *C. auris* isolates resistant to FLU [57,58]. According to the mechanisms of action of farnesol and its derivatives on fungal cells, the exogenous farnesol leads to alterations in the cell membrane by inhibiting the synthesis of ergosterol [59], which is the possible mechanism of farnesol in combination therapy. The azole drugs inhibit the biosynthesis of ergosterol by blocking the action of cytochrome P450-dependent enzyme 14-alpha-demethylase, resulting in the disruption of the plasma membrane, which also explains the synergistic effect of farnesol and azoles in our study [60]. Therefore, farnesol inhibition of the ergosterol biosynthetic pathway might decrease the levels of the intermediates. Hence, its combination with VCZ may result in an indifferent interaction. Farnesol also shows antineoplastic activity by the downregulation of cell proliferation and enhancement of apoptosis in some human cancer cell lines, such as breast cancer, lung cancer, and multiple myeloma, with some known mechanisms [17,61]. Although farnesol has apoptotic influences and chromosomal damage in cancer cell lines in certain concentrations, it has no apoptotic effect on the healthy human lung epithelial BEAS-2B cell line [60]. In line with these conclusions, our flow cytometry findings indicate that farnesol has no apoptosis activity in the SW480 cell line (a colon cancer line), indicating it to be a safe agent for mammalian cells. In agreement with our findings, in a study by Cernakova et al. [58], in 2018, while farnesol (200 µM) effectively reduced yeast to hyphae transition in a dual biofilm of *C. albicans* and *Streptococcus mutans*, it did not exhibit a cytotoxicity effect on larvae *Galleria mellonella*.

As other studies, our work presents limitations. Due to restricted research fundings it was not possible to perform a flow cytometry test for the combination of farnesol/drug. Additionally, this study focused on planktonic cultures and not biofilms, which are important biological forms for persistent and/or drug-resistant infections. Therefore, future studies are needed to test the validity of the approach presented in the current article. Additionally, although we evaluated clinical VVC cases, the cytotoxicity assay was performed with a cell line derived from colon cancerous cells, which may present some variations compared with vaginal cells. Therefore, additional studies are needed to determine if there are any cell-specific effects of the test compounds used in the current study.

We stress that the FICI interpretation does not only assess the decrease of MIC. It is possible to verify if the combination can or cannot reverse the resistance/tolerance effect of a drug in a strain (as performed in this study and others). By saying this, we would like to highlight that this kind of approach is particularly important in low-income countries (such as Iran), where the access to novel drugs is extremely difficult, so the combination of common drugs into cheap compounds (natural compounds) can be a very important clinical answer. To our knowledge, this is the first study conducted directly on VVC clinical isolates for the purpose of selecting a combination of farnesol and commercial drugs to obtain a basic protocol for future studies and lead to practical solutions in similar populations. Finally, in a future work, it would be interesting to evaluate the expression level of the efflux pumps in non-*albicans Candida* species to check the background of farnesol-related tolerance/resistance reversion.

## 5. Conclusions

A combination of farnesol with common antifungal drugs might enhance the activity of fluconazole and itraconazole in resistant isolates, with a significant decrease in MIC, suggesting that it might be a promising antifungal agent. One point worth highlighting is the necessity of further studies to uncover the role of farnesol in the sterol biosynthesis and gene expression that contribute to the regulation of this pathway and how it interferes with cells.

## Figures and Tables

**Table 1:** Primers employed in the multiplex PCR amplification.

*Candida* Species	Sequences (5′->3′)	Amplicons
*C. albicans*	F^5′^AGATTATTGCCATGCCCTGAG^3′^ R^5′^CCATGTCGAACGTAGCGTATGC^3′^	606 bp
*C. glabrata*	F^5′^ACCGTGCTTGCCTCTACA^3′^ R^5′^GACATCTGAGCCTCGTCTGA^3′^	212 bp
*C. tropicalis*	F^5′^AGAACAAGAAAACAGTGAAGCAA^3′^ R^5′^CCATGTCGAACGTAGCGTATGC^3^	126 bp
*C. parapsilosis*	F^5′^TACACCAAGCGACTCAGC^3′^ R^5′^ACCAGCTGCTTTGACTTG^3′^	490 bp
*C. krusei*	F^5′^GGCGTTGTCCATCCAATG^3′^ R^5′^CAGGAGAATTGCTGTTCCC^3′^	1159 bp
*C. dubliniensis*	F^5′^GTCGGACATATACCTCCAACTC^3′^ R^5′^CCATGTCGAACGTAGCGTAT^3′^	718 bp

**Table 2:** Antifungal susceptibility pattern of *Candida* species against antifungal drugs (CLSI M27-A3/S4).

*Candida* Species	Antifungal Drug	Sensitive (S)		Dose-Dependent		Resistance (R)	
		n	%	N	%	n	%
*C. albicans* n = 35	FLU	11	31.4	1	2.1	23	65.7
	ITZ	18	51.4	-	-	17	48.5
	VOR	17	48.5	-	-	18	51.4
	AMB	35	100	-	-	-	-
	CTZ	13	37.1	-	-	22	62.8
*C. glabrata* n = 39	FLU	9	23	2	5.1	28	71.8
	ITZ	16	41	-	-	23	59
	VOR	14	35.9	-	-	25	64.1
	AMB	35	89.7	-	-	4	10.2
	CTZ	13	33.3	-	-	26	66.6
*C. parapsilosis* n = 3	FLU	2	66.6	-	-	1	33.3
	ITZ	3	100	-	-	-	-
	VOR	2	66.6	-	-	1	33.3
	AMB	1	33.3	-	-	2	66.6
	CTZ	1	33.3	-	-	2	66.6

**Table 3:** Minimum inhibitory concentrations (MICs) of farnesol and antifungals alone and in combinations with farnesol against the resistant *Candida* isolates.

Isolates	Median MIC Values				Interaction Analysis	
	MIC Alone		MIC in Combination		Median FICI	Type of Interaction
	FLU (µg/L)	FAR (µM)	FLU (µg/L)	FAR (µM)		
*C. albicans*	64 (8–64)	300	8 (2–8)	150	0.5	**Synergy**
*C. glabrata*	64 (8–64)	300	8 (2–16)	300	0.9	Indifferent
*C. parapsilosis*	32 (8–32)	300	4 (2–8)	150	0.35	Synergy
	ITRA (µg/L)	FAR (µM)	ITRA (µg/L)	FAR (µM)		
*C. albicans*	8 (1–8)	300	4 (1–8)	150	0.5	**Synergy**
*C. glabrata*	8 (2–8)	300	8 (2–8)	300	1.01	Indifferent
*C. parapsilosis*	8 (2–8)	300	4 (1–4)	150	0.25	**Synergy**
	VOR (µg/L)	FAR (µM)	VOR (µg/L)	FAR (µM)		
*C. albicans*	16 (2–16)	300	8 (1–8)	150	0.75	Indifferent
*C. glabrata*	16 (2–16)	300	8 (2–16)	300	0.75	Indifferent
*C. parapsilosis*	8 (2–16)	300	4 (1–4)	150	0.5	**Synergy**
	AmB (µg/L	FAR (µM)	AmB (µg/L)	FAR (µM)		
*C. albicans*	2 (0.031–2)	300	2 (0.031–2)	150	–	–
*C. glabrata*	2 (0.031–2)	300	1 (0.031–2)	300	1.25	Indifferent
*C. parapsilosis*	2 (0.031–2)	300	1 (0.031–2)	150	0.35	**Synergy**
	CTZ (µg/L)	FAR (µM)	CTZ (µg/L)	FAR (µM)		
*C. albicans*	16 (2–16)	300	4 (1–4)	150	1.75	Indifferent
*C. glabrata*	16 (2–16)	300	8 (2–16)	300	0.9	Indifferent
*C. parapsilosis*	8 (2–16)	300	2 (0.5–4)	150	1.25	Indifferent

## Data Availability

Not applicable.

## References

[1] Denning D.W., Bromley M.J. (2015). How to Bolster the Antifungal Pipeline: Few Drugs Are Coming to Market, but Opportunities for Drug Development Exist. Science.

[2] Eckert L., Hawes S., Stevens C., Koutsky L., Eschenbach D., Holmes K. (1998). Vulvovaginal Candidiasis: Clinical Manifestations, Risk Factors, Management Algorithm. Obstet. Gynecol..

[3] Pirotta M.V., Garland S.M. (2006). Genital Candida Species Detected in Samples from Women in Melbourne, Australia, before and after Treatment with Antibiotics. J. Clin. Microbiol..

[4] Denning D.W., Kneale M., Sobel J.D., Rautemaa-Richardson R. (2018). Global burden of recurrent vulvovaginal candidiasis: A systematic review. Lancet Infect. Dis..

[5] Lema V.M. (2017). Recurrent Vulvo-Vaginal Candidiasis: Diagnostic and Management Challenges in a Developing Country Context. Obstet. Gynecol. Int. J..

[6] Lírio J., Giraldo P.C., Amaral R.L., Sarmento A.C.A., Costa A.P.F., Gonçalves A.K. (2019). Antifungal (Oral and Vaginal) Therapy for Recurrent Vulvovaginal Candidiasis: A Systematic Review Protocol. BMJ Open.

[7] Chew S.Y., Thian L., Than L. (2016). Vulvovaginal candidosis: Contemporary challenges and the future of prophylactic and therapeutic approaches. Mycoses.

[8] Li C., Xu Z., Liu S., Huang Y., Duan W., Wei X. (2021). In Vivo Antifungal Activities of Farnesol Combined with Antifungal Drugs against Murine Oral Mucosal Candidiasis. Biofouling.

[9] Sobel J., Sobel R. (2018). Current Treatment Options for Vulvovaginal Candidiasis Caused by Azole-Resistant Candida Species. Expert Opin. Pharmacother..

[10] Costa A.F., Silva L.C., Amaral A.C. (2021). Farnesol: An approach on biofilms and nanotechnology. Med. Mycol..

[11] Brilhante R.S.N., de Lima R.A.C., Caetano E.P., Leite J.J.G., Castelo-Branco D.S.C.M., Riberio J.F., Bandeira T.D.J.P.G., Cordeiro R.D.A., Monteiro A.J., Sidrim J.J.C. (2013). Effect of Farnesol on Growth, Ergosterol Biosynthesis, and Cell Permeability in Coccidioides Posadassi. Antimicrob. Agent Chemother..

[12] Wang X., Wang Y., Zhou Y., Wei X. (2014). Farnesol Induces Apoptosis-like Cell Death in the Pathogenic Funus Aspergillus Flavus. Mycologia.

[13] Delmondes G.D.A., Santiago Lemos I.C., Dias D.D.Q., Da Cunha G.L., Araújo I.M., Barbosa R., Coutinho H.D.M., Felipe C.F.B., Barbosa-Filho J.M., De Lima N.T.R. (2020). Pharmacological Applications of Farnesol (C15H26O): A Patent Review. Expert Opin. Ther. Pat..

[14] Ramage G., Saville S., Wickes B., Lopez-Ribot J. (2002). Inhibition of Candida Albicans Biofilm Formation by Farnesol, a Quorum-Sensing Molecule. Appl. Environ. Microbiol..

[15] Sebaa S., Boucherit-Otmani Z., Courtois P. (2019). Effects of Tyrosol and Farnesol on Candida Albicans Biofilm. Mol. Med. Rep..

[16] Rodrigues C.F., Černáková L. (2020). Farnesol and Tyrosol: Secondary Metabolites with a Crucial Quorum-Sensing Role in Candida Biofilm Development. Genes.

[17] Polke M., Leonhardt I., Kurzai O., Jacobsen I.D. (2018). Farnesol Signalling in *Candida Albicans*—More than Just Communication. Crit. Rev. Microbiol..

[18] Katragkou A., Mccarthy M., Alexander E.L., Antachopoulos C., Meletiadis J., Jabra-rizk M.A., Petraitis V., Roilides E., Walsh T.J. (2015). In Vitro Interactions between Farnesol and Fluconazole, Amphotericin b or Micafungin against Candida Albicans Biofilms. J. Antimicrob. Chemother..

[19] Onder S., Oz Y. (2021). In Vitro Effects of Farnesol Alone and in Combination with Antifungal Drugs against Aspergillus Clinical Isolates. Med. Mycol. J..

[20] Bozó A., Domán M., Majoros L., Kardos G., Varga I., Kovács R. (2016). The in Vitro and in Vivo Efficacy of Fluconazole in Combination with Farnesol against Candida Albicans Isolates Using a Murine Vulvovaginitis Model. J. Microbiol..

[21] Spitzer M., Robbins N., Wright G.D. (2017). Combinatorial Strategies for Combating Invasive Fungal Infections. Virulence.

[22] Gong Y., Liu W., Huang X., Hao L., Li Y., Sun S. (2019). Antifungal Activity and Potential Mechanism of N-Butylphthalide Alone and in Combination with Fluconazole against Candida Albicans. Front. Microbiol..

[23] Rodrigues C.F., Boas D.V., Haynes K., Henriques M. (2018). The MNN2 Gene Knockout Modulates the Antifungal Resistance of Biofilms of Candida Glabrata. Biomolecules.

[24] Arastehfar A., Fang W., Pan W., Liao W., Yan L., Boekhout T. (2018). Identification of Nine Cryptic Species of *Candida albicans*, *C. glabrata*, and *C. parapsilosis* Complexes Using One-Step Multiplex PCR. BMC Infect. Dis..

[25] Nikoomanesh F., Roudbarmohammadi S., Khoobi M., Haghighi F., Roudbary M. (2019). Design and Synthesis of Mucoadhesive Nanogel Containing Farnesol: Investigation of the Effect on HWP1, SAP6 and Rim101 Genes Expression of Candida Albicans in Vitro. Artif. Cells Nanomed. Biotechnol..

[26] Zare-Bidaki M., L Maleki A., Ghanbarzadeh N. (2022). Expression pattern of drug-resistance genes *ERG11* and *TAC1* in *Candida albicans* Clinical isolates. Mol. Biol. Rep..

[27] Clinical and Laboratory Standards Institute (2008). Reference Method for Broth Dilution Antifungal Susceptibility Testing of Yeasts.

[28] Nikoomanesh F., Roudbarmohammadi S., Bashardoust B., Zareei M. (2018). Effect of Farnesol on Responsive Gene Expressions in Hyphal Morphogenesis Transformation of Candida Albicans. Infect. Epidemiol. Microbiol..

[29] Meletiadis J., Mouton J.W., Meis J.F., Verweij P.E. (2003). In vitro drug interaction modeling of combinations of azoles with terbinafine against clinical Scedosporium prolificans isolates. Antimicrob. Agents Chemother..

[30] Alipour R., Fatemi A., Alsahebfosul F., Andalib A., Pourazar A. (2020). Autologous Plasma versus Fetal Calf Serum as a Supplement for the Culture of Neutrophils. BMC Res. Notes.

[31] Kumar S., Kumar A., Roudbary M., Mohammadi R., Černáková L. (2022). Overview on the Infections Related to Rare Candida Species. Pathogens.

[32] Roudbary M., Roudbarmohammadi S.H., Bakhshi B., Farhadi Z., Nikoomanesh F. (2013). Identification of Candida Species Isolated Form Iranian Women Eith Vaginal Candidasis by PCR-RFLP Method. Eur. J. Exp. Biol..

[33] Alves A.M.C.V., Cruz-Martins N., Rodrigues C.F. (2022). Marine Compounds with Anti-Candida Sp. Activity: A Promised “Land” for New Antifungals. J. Fungi.

[34] Zeng X., Zhang Y., Zhang T., Xue Y., Xu H., An R. (2018). Risk Factors of Vulvovaginal Candidiasis among Women of Reproductive Age in Xi’an: A Cross-Sectional Study. Biomed. Res. Int..

[35] Arendrup M.C., Patterson T.F. (2017). Multidrug-Resistant Candida: Epidemiology, Molecular Mechanisms, and Treatment. J. Infect. Dis..

[36] Rodrigues C.F., Gonçalves B., Rodrigues M.E., Silva S., Azeredo J., Henriques M. (2017). The Effectiveness of Voriconazole in Therapy of Candida Glabrata’s Biofilms Oral Infections and Its Influence on the Matrix Composition and Gene Expression. Mycopathologia.

[37] Nyirjesy P., Brookhart C., Lazenby G., Schwebke G., Sobel J.D. (2022). Vulvovaginal Candidiasis: A Review of the Evidence for the 2021 Centers for Disease Control and Prevention of Sexually Transmitted Infections Treatment Guidelines. Clin. Infect. Dis..

[38] Maraki S., Mavromanolaki V.E., Stafylaki D., Nioti E., Hamilos G., Kasimati A. (2019). Epidemiology and antifungal susceptibility patterns of Candida isolates from Greek women with vulvovaginal candidíases. Mycoses.

[39] Guzel A.B., Ilkit M., Burgut R., Urunsak I.F., Ozgunen F.T. (2011). An Evaluation of Risk Factors in Pregnant Women with Candida Vaginitis and the Diagnostic Value of Simultaneous Vaginal and Rectal Sampling. Mycopathologia.

[40] Bitew A., Abebaw Y. (2018). Vulvovaginal Candidiasis: Species Distribution of Candida and Their Antifungal Susceptibility Pattern. BMC Womens Health.

[41] Arastehfar A., Kargar M.L., Mohammadi S.R., Roudbary M., Ghods N., Haghighi L., Daneshnia F., Tavakoli M., Jafarzadeh J., Hedayati M.T. (2021). A High Rate of Recurrent Vulvovaginal Candidiasis and Therapeutic Failure of Azole Derivatives among Iranian Women. Front. Microbiol..

[42] Mohammadi-Ghalehbin B., Javanpour Heravi H., Arzanlou M., Sarvi M. (2017). Prevalence and Antibiotic Resistance Pattern of Candida Spp. Isolated from Pregnant Women Referred to Health Centers in Ardabil, Iran. J. Ardabil. Univ. Med. Sci..

[43] Cowen L.E., Sanglard D., Howard S.J., Rogers P.D., Perlin D.S. (2015). Mechanisms of Antifungal Drug Resistance. Cold Spring Harb. Perspect. Med..

[44] Sustr V., Foessleitner P., Kiss H., Farr A. (2020). Vulvovaginal Candidosis: Current Concepts, Challenges and Perspectives. J. Fungi.

[45] Fakhim H., Vaezi A., Dannaoui E., Chowdhary A., Nasiry D., Faeli L., Meis J.F., Badali H. (2018). Comparative virulence of Candida auris with Candida haemulonii, Candida glabrata and Candida albicans in a murine model. Mycoses.

[46] Hornby J.M., Jensen E.C., Lisec A.D., Tasto J.J., Jahnke B., Shoemaker R., Dussault P., Nickerson K.W. (2001). Quorum Sensing in the Demorphic Fungus Candida Albicans Is Mediated by Farnesol. Appl. Environ. Microbiol..

[47] Sachivkina N., Podoprigora I., Bokov D. (2021). Morphological characteristics of Candida albicans, Candida krusei, Candida guilliermondii, and Candida glabrata biofilms, and response to farnesol. Vet. World.

[48] Yu L.H., Wei X., Ma M., Chen X.J., Xu S.B. (2012). Possible inhibitory molecular mechanism of farnesol on the development of fluconazole resistance in Candida albicans biofilm. Antimicrob. Agents Chemother..

[49] Nagy F., Vitális E., Jakab Á., Borman A.M., Forgács L., Tóth Z., Majoros L.K.R. (2020). In Vitro and in Vivo Effect of Exogenous Farnesol Exposure Against Candida Auris. Front. Microbiol..

[50] Meletiadis J., Pournaras S., Roilides E., Walsh T.J. (2010). Defining fractional inhibitory concentration index cutoffs for additive interactions based on self-drug additive combinations, Monte Carlo simulation analysis, and in vitro-in vivo correlation data for antifungal drug combinations against Aspergillus fumigatus. Antimicrob. Agents Chemother..

[51] Décanis N., Tazi N., Correia A., Vilanova M., Rouabhia M. (2011). Farnesol, a Fungal Quorum-Sensing Molecule Triggers Candida Albicans Morphological Changes by Downregulating the Expression of Different Secreted Aspartyl Proteinase Genes. Open Microbiol. J..

[52] Rodrigues C.F., Alves D.F., Henriques M. (2018). Combination of Posaconazole and Amphotericin b in the Treatment of Candida Glabrata Biofilms. Microorganisms.

[53] Cordeiro R.A., Teixeira C.E.C., Brilhante R.S.N., Castelo-Branco D.S.C.M., Paiva M.A.N., Giffoni Leite J.J., Lima D.T., Monteiro A.J., Sidrim J.J.C., Rocha M.F.G. (2013). Minimum Inhibitory Concentrations of Amphotericin B, Azoles and Caspofungin against Candida Species Are Reduced by Farnesol. Med. Mycol..

[54] Xia J., Qian F., Xu W., Zhang Z., Wei X. (2017). In Vitro Inhibitory Effects of Farnesol and Interaction between Farnesol and Antifungals against Biofilm of C Andida Albicans Resistance Strains. Biofouling.

[55] Bezerra C.F., de Alencar Júnior J.G., de Lima Honorato R., dos Santos A.T.L., Pereira da Silva J.C., Gusmão da Silva T., Leal A.L.A.B., Rocha J.E., de Freitas T.S., Tavares Vieira T.A. (2020). Antifungal Activity of Farnesol Incorporated in Liposomes and Associated with Fluconazole. Chem. Phys. Lipids.

[56] Sharma M., Prasad R. (2011). The Quorum-Sensing Molecule Farnesol Is a Modulator of Drug Efflux Mediated by ABC Multidrug Transporters and Synergizes with Drugs in Candida Albicans. Antimicrob. Agents Chemother..

[57] Černáková L., Dižová S., Gášková D., Jančíková I., Bujdáková H. (2019). Impact of Farnesol as a Modulator of Efflux Pumps in a Fluconazole-Resistant Strain of Candida Albicans. Microb. Drug Resist..

[58] Dekkerová J., Černáková L., Kendra S., Borghi E., Ottaviano E., Willinger B., Bujdáková H. (2022). Farnesol Boosts the Antifungal Effect of Fluconazole and Modulates Resistance in Candida Auris through Regulation of the CDR1 and ERG11 Genes. J. Fungi.

[59] Jabra-Rizk M.A., Shirtliff M., James C., Meiller T. (2006). Effect of Farnesol on Candida Dubliniensis Biofilm Formation and Fluconazole Resistance. FEMS Yeast Res..

[60] Öztürk B.Y., Feyzullazade N., Dağ I., Şengel T. (2022). The investigation of in vitro effects of farnesol at different cancer cell lines. Microsc. Res. Tech..

[61] Shi Y., Zhu Y., Fan S., Vitagliano A., Liu X., Liao Y., Liang Y., Vitale S.G. (2019). Clinical Characteristics and Antifungal Susceptibility of Candida Nivariensis from Vulvovaginal Candidiasis. Gynecol. Obstet. Investig..

